# Association of vitamin D deficiency and insufficiency with uncontrolled type 1 diabetes Mellitus among Saudi pediatric patients; a hospital-based retrospective study

**DOI:** 10.3389/fped.2024.1479815

**Published:** 2025-01-07

**Authors:** Salman Almansour, Abdullrahman Alsalamah, Mohammad Almutlaq, Ahmed Sheikh, Hamdan Z. Hamdan, Abdullah Al-Nafeesah, Ashwaq AlEed, Ishag Adam, Osama Al-Wutayd

**Affiliations:** ^1^ Department of Pediatrics, College of Medicine, Qassim University, Buraydah, Saudi Arabia; ^2^ Department of Pediatrics, King Saud Hospital, Unaizah, Saudi Arabia; ^3^ Diabetic Center, King Saud Hospital, Unaizah, Saudi Arabia; ^4^ Department of Pathology, College of Medicine, Qassim University, Buraydah, Saudi Arabia; ^5^ Department of Obstetrics and Gynecology, College of Medicine, Qassim University, Buraydah, Saudi Arabia; ^6^ Department of Family and Community Medicine, College of Medicine, Qassim University, Buraydah, Saudi Arabia

**Keywords:** prevalence, diabetes Mellitus, child, vitamin D, HbA1c, glycemic control, thyroid function

## Abstract

**Background:**

The association between 25-hydroxy-vitamin D [25(OH)D] levels and glycemic control in pediatric patients with type 1 diabetes mellitus (T1DM) is unclear. In this study, we aimed to investigate the association between 25(OH)D levels and glycemic control in Saudi pediatric patients' with T1DM in a region that is sunny year-round.

**Materials and methods:**

A retrospective study was conducted in the Pediatric Department of King Saud Hospital in Unaizah, Saudi Arabia. A total of 218 children with T1DM were enrolled in the study and grouped according to their glycated hemoglobin (HbA_1C_) levels into the controlled T1DM (HbA_1C_ ≤ 7.5%) and the uncontrolled T1DM (HbA_1C_ > 7.5%). Their 25(OH)D levels and thyroid function were measured using standard methods.

**Results:**

Of the 218 children in this study, 182 (83.5%) had uncontrolled T1DM, while only 36 (16.5%) had controlled T1DM. The median (interquartile range) of 25(OH)D levels was significantly lower in the uncontrolled T1DM group compared with the controlled group [45.4 (31.2–59.7) nmol/L vs. 56.1 (37.5–77.6) nmol/L; *p* = 0.007], respectively. Vitamin D deficiency (<50.0 nmol/L) and insufficiency (50–74 nmol/L) were detected in 55.0% and 31.1% of all the enrolled children, respectively. Vitamin D deficiency was detected in 86.6% of the uncontrolled T1DM patients and in 16.5% of the controlled T1DM patients (*p* = 0.012). The multivariable analysis showed that both vitamin D deficiency [adjusted odds ratio (aOR) = 2.92, *p* = 0.048] and insufficiency [aOR = 3.17, *p* = 0.042] were risk factors for uncontrolled diabetes.

**Conclusion:**

Vitamin D deficiency was highly prevalent in the studied group. Both vitamin D deficiency and insufficiency are associated with uncontrolled T1DM. Further study is needed.

## Introduction

Type 1 diabetes mellitus (T1DM) in children is a chronic endocrinological disease that is characterized by hyperglycemia due to either relative or complete insulin deficiency (1). It is a worldwide concern, with its global incidence increasing annually by up to 4%. Saudia Arabia is no exception to this international pattern; the World Health Organization (WHO) ranks Saudi Arabia as the 7th leading country in T1DM prevalence worldwide (2).

T1DM can be described etiologically as an autoimmune disease in which self-immune cells target pancreatic cells. Hypothyroidism, which often presents as autoimmune hypothyroidism, has been identified as a contributing factor in the development of T1DM. It may proceed the development of diabetes or come later (3).

Vitamin D is known primarily for its crucial role in calcium metabolism and bone health. Its deficiency leads to rickets in children and osteomalacia in adults (4). It acts as a pro-hormone, exerting its action through a specific receptor expressed in various target tissues. The rapidly emerging and continuous research on vitamin D related functions has revealed that the biological and metabolic role of vitamin D is not limited to the skeletal tissues only but extends to other body tissues, including the pancreas and immune cells (5).

A growing body of evidence has pointed to the association between 25-hydroxy-vitamin D [25(OH)D] levels and T1DM (6, 7). Experimental evidence has shown that in the pancreatic tissues, insulin secretion is affected by 25(OH)D levels (8). Moreover, many studies have reported that the 25(OH)D levels is inversely correlated with the level of glycosylated hemoglobin (HbA_1C_), which indicates that 25(OH)D affects the level of glycemic control among children with T1DM (9, 10). Furthermore, interventional studies have shown that vitamin D supplementation improves the *β*-cell function (11) and the glycemic controls (12). Conversely, some studies have found no association between 25(OH)D levels and glycemic controls (13, 14). It has been reported that, the glycemic control of T1DM can be affected by the levels of thyroid hormones (15). Hyperthyroidism accelerates the catabolism of the insulin hormone, precipitating hyperglycemia and consequently deteriorates the levels of glycemic control (15). Moreover, an inverse correlation was observed between the thyroid stimulating hormone (TSH) and HbA_1C_ (16). Additionally, a recent study reported that children with T1DM who have hypothyroidism have higher levels of HbA_1c_ compare with patients without hypothyroidism (17). This study aimed to investigate the factors that may affect the HbA_1c_ level in pediatric patients with T1DM. These factors include 25(OH)D levels, TSH and Free T4 (FT4) levels, as well as body mass index. To the best of our knowledge, this is the first study that investigated these factors in association with T1DM with children in our region of Qassim in Saudi Arabia. The findings of this study is expected to help clinicians and caregivers to enhance the glycemic control of their pediatric patients.

## Materials and methods

This study was a retrospective cross-sectional study conducted between March 2020 and February 2023 in the Pediatrics Department of King Saud Hospital in Unaizah, Qassim region in Saudi Arabia. It included Saudi children aged 2–17 years who had been diagnosed with T1DM, whose 25(OH)D level was recently measured, and who had regular follow-ups for six months. T1DM was diagnosed based on the guidelines of the American Diabetic Association (18). Patients with HbA_1C_ ≤ 7.5% were regarded as having controlled diabetes, and those with HbA_1C_ > 7.5%, as having uncontrolled diabetes (19).

Sociodemographic and anthropometric data were collected from the patients using a structured questionnaire. The data included the patients' age, weight, and height, as well as clinical and biochemical data, such as vitamin D, HbA_1C_, and thyroid hormone function levels. The body mass index (BMI) was calculated as the weight in kilograms divided by the squared height in meters. Then, the calculated BMI was converted into the BMI *z*-score using the WHO international reference chart for children (20). The BMI z-score was interpreted as follows: severe thinness (SD <−3, thinness-underweight (SD − 2 to − 3), normal (SD − 2 to + 1), overweight (SD + 1 to + 2), and obesity (SD > + 2).

### Laboratory measurements

The 25(OH)D level was measured using enzyme-linked immunosorbent assay (ELISA) kits (Euroimmun, Lubeck, Germany) according to the manufacturer's instructions. Six calibrators were used with a concentration range between 0 and 120 ng/ml, for which the manufacturer-recommended quality measures were strictly followed. A plasma 25(OH)D level of >75 nmol/L was categorized as adequate; 50–74 nmol/L, insufficient; and <50.0 nmol/L, deficient (21). The blood levels of serum thyroid profile (FT4 and TSH) levels were measured using the immunoassay analyser AIA 360 (Tosoh Bioscience, San Francisco, CA, USA), guided by the manufacturer's instructions.

### Sample size calculation

The sample size was calculated using the OpenEpi calculator (22), guided by the reported mean 25(OH)D concentration of 35.15 nmol/L among Saudi children with diabetes (14). We assumed that the mean 25(OH)D concentration of the children with uncontrolled T1DM would be 28.0 nmol/L and that would differ from the mean 25(OH)D concentration of the children with controlled T1DM by 7 nmol/L. We found that to achieve 80% power and 5% precision, a sample size of 110 in each group was needed.

### Statistical analysis

The data were entered into a computer and cleaned using the Microsoft Excel software. Statistical analysis was performed using the STATA software (version 16). The patients were divided into the following two groups based on their glycemic control level: into the controlled diabetes group for the patients with an HbA_1c_ ≤ 7.5% and the uncontrolled diabetes group for the patients with HbA_1c_ > 7.5%. The Kolmogorov-Smirnov test was used to determine the distribution of the data. Normally distributed continuous data were expressed as means [i.e., standard deviations (SDs)], and non-normally distributed data were expressed as medians [i.e., interquartile ranges (IQRs)]. Categorical data were expressed as numbers (%).

A multivariable analysis model was constructed to determine the factors associated with uncontrolled T1DM in children. Variables with a *p* value < 0.25 in the bivariable analysis were considered in the model. Two models were constructed in multivariable analysis, the first one includes 25(OH)D level as a categorical variable and the second one as a continuous variable to avoid multicollinearity. The crude odds ratios (cORs) and adjusted odds ratios (aORs), along with the 95% confidence intervals (95% CIs), were reported, and *p* values < 0.05 were considered statistically significant.

## Results

A total of 218 children diagnosed with T1DM were included in this study, of whom 117 (53.7%) were boys. More than half (56.4%) of the children were underweight at the time of their T1DM diagnosis, but this figure has declined to 47.3% currently. In contrast, 21.6% and 13.3% of the children were overweight and obese, respectively, at the time of their T1DM diagnosis, but these figures have risen to 22.9% and 27.9%, respectively, at present. Fifty-five percent of all the children were deficient in vitamin D, and 31.1% had insufficient vitamin D. The overall median IQR age at diagnosis was 9 (5–11) years, and the current age (IQR) was 12 (9–17) years. The median (IQR) of the TSH, FT4, and 25(OH)D levels in all the studied samples are shown in Table 1.

**Table 1:** Sociodemographic and clinical characteristics of the pediatrics participant with diabetes mellitus type 1.

Variables	Frequency	%
Gender		
Male	117	53.7
Female	101	46.3
BMI *Z* score at diagnosis		
Underweight	123	56.4
Normal	19	8.7
Overweight	47	21.6
Obese	29	13.3
BMI *Z* score—current		
Underweight	103	47.3
Normal	4	1.8
Overweight	50	22.9
Obese	61	27.9
Vitamin D status		
Sufficiency	30	13.7
Insufficiency	68	31.1
Deficiency	120	55.0
	Median	IQR
Current age, years	12	9–17
Age at diagnosis, years	9	5–11
HbA_1C_,%	9.2	8–10.9
TSH, *μ*IU/ml	2.6	1.6–4.2
FT4, Pmol/L	16.2	14.4–18.3
25(OH)D levels, nmol/L	46.3	32–61.4

### Bivariate and multivariable analyses of factors associated with uncontrolled diabetes mellitus

Of the 218 children included in this study, 182 (83.5%) had uncontrolled T1DM (HbA_1C_ ≥ 7.5%), and only 36 (16.5%) had controlled T1DM (HbA_1C_ ≤ 7.5%). The bivariable analysis showed that only the current age (cOR = 1.09; *p* value = 0.025), FT4 level (cOR = 0.88; *p* value = 0.038), median 25(OH)D level (cOR = 0.98; *p* value = 0.007), vitamin D deficiency (cOR = 3.25; *p* value = 0.012), and vitamin D insufficiency (cOR = 2.90; *p* value = 0.039) were significantly associated with uncontrolled T1DM (Table 2). In the multivariable analysis, the model results revealed that the aORs of vitamin D deficiency (2.92; *p* = 0.048) and insufficiency (3.17; *p* = 0.042) were significant risk factors for uncontrolled T1DM. The other factors in the model, such as the BMI, child age, and TSH and FT4 levels, were not statistically significant (Table 3). To further confirm the association of vitamin D with the glycemic control, another model was constructed by adding the 25(OH)D levels as a median and 25(OH)D level showed a protective effect against uncontrolled diabetes (aOR = 0.98; *p* = 0.048), other factors remained not statistically significant, Table 3.

**Table 2:** Bivariate analysis and crude odds ratio (cOR) of the factors possibly associated with uncontrolled diabetes.

	Uncontrolled diabetes HbA_1C_ > 7.5%	Controlled diabetes HbA_1C_ ≤ 7.5%	cOR (95% CI)	*p*-Value
	*N* = 182 (83.5%)	*N* = 36 (16.5%)		
	Frequency (%)	Frequency (%)		
Gender				
Male	96 (82.1)	21 (17.9)	0.80 (0.39–1.64)	0.540
Female	86 (85.2)	15 (14.9)	Reference	
BMI z score at diagnosis				
Underweight	14 (73.7)	5 (26.3)	0.64 (0.21–1.97)	0.440
Normal	100 (81.3)	23 (18.7)	Reference	
Overweight	44 (93.6)	3 (6.4)	3.37 (0.96–11.8)	0.057
Obese	24 (82.8)	5 (17.2)	1.10 0.38–3.20	0.855
BMI z score—current				
Underweight	2 (50)	2 (50)	0.20 (0.03–1.50)	0.117
Normal	86 (83.5)	17 (16.5)	Reference	
Overweight	45 (90)	5 (10)	1.78 (0.62–5.14)	0.287
Obese	49 (80.3)	12 (19.7)	0.81 (0.36–1.82)	0.608
Vitamin D classification				
Sufficient	20 (66.6)	10 (33.3)	Reference	
Insufficient	58 (85.2)	10 (14.7)	2.9 (1.052–7.987)	0.039*
Deficiency	104 (86.6)	16 (16.5)	3.25 (1.290–8.185)	0.012*
	Median (IQR)	Median (IQR)	cOR (95% CI)	
Current age, years	12.5 (9–17)	9 (7–15)	1.09 (1.01–1.18)	0.025*
Age at diagnosis, years	9 (5–12)	8 (5.8–11)	1.01 (0.92–1.10)	0.910
TSH, μIU/ml	2.6 (1.6–4.6)	2.17 (1.3–3.3)	1.13 (0.96–1.32)	0.123
FT4, Pmol/L	16.1 (14.3–18.2)	17 (15.4–19.2)	0.88 (0.78–0.99)	0.038*
25(OH)D levels, nmol/L	45.4 (31.2–59.7)	56.1 (37.5–77.6)	0.98 (0.97–0.99)	0.007*

Significant *p*-value,*.

**Table 3:** Multivariable analysis of the factors associated with uncontrolled diabetes.

	aOR (95% CI)	*p*-Value
BMI z score at diagnosis		
Underweight	0.83 (0.22–3.22)	0.796
Normal	Reference	
Overweight	3.5 (0.92–13.45)	0.066
Obese	1.19 (0.34–4.25)	0.783
BMI z score—current		
Underweight	0.28 (0.03–3.01)	0.299
Normal	Reference	
Overweight	0.91 (0.27–3.05)	0.299
Obese	0.40 (0.13–1.23)	0.109
Current age	1.10 (0.99–1.23)	0.065
TSH	1.20 (0.98–1.49)	0.079
FT4	0.91 (0.80–1.05)	0.226
Vitamin D insufficiency	3.17 (1.04–9.68)	0.042*
Vitamin D deficiency	2.92 (1.01–8.50)	0.048*
25(OH)D levels, nmol/L	0.98 (0.96–0.99)	0.048^‡^

Significant *p*-value, *.

Entered into another model, ^‡.^

## Discussion

The main finding of the current study is that vitamin D deficiency and insufficiency are considered risk factors for uncontrolled T1DM in children. This finding aligns with the findings from ALkharashi study on Saudi children with T1DM, as well as with those of studies from Kuwait and Spain (23–25). This finding is partially explained by the molecular function of in body cells, including those in the pancreas and immune systems. T1DM is an autoimmune disease characterized by immune cell infiltration into the pancreatic *β*-cells (1). The major immune cells involved in this infiltration are cytotoxic and regulatory T cells (26). It is worth mentioning that the vitamin D receptor is expressed in T lymphocytes, allowing it to modulate their function. Specifically, vitamin D reduces the production of Th1 cells over Th2 cells, thereby decreasing the production of pro-inflammatory cytokines, namely IL-1β, TNF-α and others (27). Noteworthy, immunomodulation therapy aimed at reducing proinflammatory cytokines such as IL-1β and TNF-α has shown a promising results in both the treatment and prevention of T1DM (27). Additionally, vitamin D regulates the production of antibodies from *β*-cells by inhibiting antigen presentation to antigen-presenting cells (28). In the case of vitamin D deficiency, the modulatory effect on T helper cells is significantly diminished, leading to increased cellular immunity and autoantibody production. In islet cells of the pancreas, vitamin D plays a protective role by downregulating gene expression related to apoptosis (29). Animal models have shown that vitamin D deficiency directly reduces the pancreas' insulin secretion in response to hyperglycemia. The insulin secretion process is well known as dependent on the calcium level, which is regulated by the availability of vitamin D (30). Correcting the 25(OH)D level appears to improve glycemic control in children with T1DM (31). Considering these premises, it is plausible that vitamin D deficiency degrades the glycemic control in children with T1DM.

The second finding of this study is that the prevalence of both vitamin D deficiency & insufficiency among all the participants was 86.1%. This figure closely matches the 81% reported by Al-Daghri in a study among mixed Saudi participant who included children (32). Despite the year-round sunny weather in this study's setting, the high prevalence of vitamin D deficiency among the study participants can be attributed to the hot environmental conditions in the Gulf region (33), which reduce outdoor activities and direct sun exposure (34). Another possible factor is the high percentage of overweight children among our study participants at their T1DM diagnosis (20%), as it has been reported that overweight children are more prone to vitamin D deficiency (35). Researchers have hypothesized that since vitamin D is fat-soluble, its bioavailability decreases due to its distribution and sequestration in adipose tissue compartment (36). However, in the multivariable model, being overweight showed borderline statistical significance, possibly due to our small sample size. Of interest, both vitamin D deficiency and obesity have been reported to exert a synergistic negative effect on glycemic control in pediatric patients (31, 37, 38). Additionally, adopting weight reduction strategies has been positively infleunce the outdoor activity, glycemic control and vitamin D levels (39).

From another perspective, recent studies have pointed out to the association between genetic variants, such as of the variants in *DHCR7* gene, and vitamin D deficiency (40, 41). This variant has been reported among Saudi families with vitamin D deficiency (42). Moreover, these variants have also been associated with vitamin D deficiency in conjunction with autoimmune T1DM (43). However, in this study, we didn't conduct a genetic analysis; otherwise, our findings would be more informative. Regardless of the genetic components of our patients, the presence of diabetes mellitus *per se* is considered an independent risk factor for vitamin D deficiency. Diabetes decreases the hepatic synthesis of 25(OH)D, which is a pre-requisite step in the vitamin D synthesis pathway, and instead, hepatocytes start to degrade the active vitamin D metabolites (44). Moreover, diabetes has been proven to inhibit the synthesis of the vitamin D binding protein, which is necessary for transporting vitamin D between body tissues (45)These factors, taken together, may explain the high prevalence of vitamin D deficiency in the current study.

In this study, we observed a statistical borderline effect of TSH on uncontrolled diabetes. Vitamin D deficiency has been well linked to hypothyroidism and autoimmune hypothyroidism (46). Moreover, among children with autoimmune thyroiditis, the 25(OH)D levels are inversely well correlated with the TSH levels (47). Furthermore, clinical trials have shown that vitamin D supplementation can normalize TSH levels in patients with hypothyroidism (48, 49). However, the exact mechanism by which vitamin D improves thyroid function is not yet fully understood.

In conclusion, high prevalence of vitamin D deficiency and insufficiency was observed in this study. Both vitamin D deficiency and insufficiency were associated with poor glycemic control among children with T1DM. The current findings are important for healthcare providers, emphasizing the needs to screen for vitamin D deficiency in children with T1DM and correcting the deficiencies when identified. Up to date, this screening is not widely implemented, therefore mor studies are needed at community levels to evaluate and consolidate this association in a large scale. However, this study had limitations that should be considered when interpreting our findings. Firstly, we did not investigate antithyroid hormone antibodies to determine the exact prevalence of autoimmune hypothyroidism. Second, since this study was a retrospective study, the participants' 25(OH)D levels at their T1DM diagnosis were not measured, so whether they already had vitamin D deficiency at their T1DM diagnosis or developed it afterward is unknown. This also limited our ability to track glycemic control in correlation with 25(OH)D level during the disease course. Additionally, due to the retrospective design of this study, causality cannot be inferred. Therefore, further study is needed with a prospective design and a larger sample size, and that will also consider autoimmune thyroid disease and investigate the genetic makeup of the patients.

## Data Availability

The raw data supporting the conclusions of this article will be made available by the authors, without undue reservation.
